# Trapline foraging by nectar-collecting hornets

**DOI:** 10.1007/s10071-025-01952-3

**Published:** 2025-04-19

**Authors:** Mathilde Lacombrade, Kristine Abenis, Charlotte Doussot, Loïc Goulefert, Kenji Nanba, Jean-Marc Bonzom, Mathieu Lihoreau

**Affiliations:** 1 Research Center on Animal Cognition (CRCA), Center of Integrative Biology (CBI), CNRS, Toulouse University, Toulouse, France; 2 Environmental Biology Division, Institute of Biological Sciences, College of Arts and Sciences, University of the Philippines Los Baños, College, Laguna, 4031 Philippines; 3 Institute of Environmental Radioactivity (IER), Fukushima University, Fukushima, Japan; 4 Nuclear Safety and Radiation Protection Authority (ARSN), PSE-ENV/SERPEN/LECO, Saint-Paul-lez-Durance, Cadarache, 13115 France; 5 Present Address: Agency for Ecological Transition (ADEME), SITESOL Department: Securing and Reconverting Polluted Wasteland, Angers, France

**Keywords:** Traplining, Spatial cognition, Artificial flowers, Spatial movement networks, *Vespa simillima*

## Abstract

Many bees, butterflies, birds, bats and primates are known to forage on familiar plant resources by visiting them in a stable and repeatable order called “traplines”. Here we report the existence of trapline foraging in wasps, the Japanese yellow hornets. We monitored the movement patterns of wild individually marked hornets collecting sucrose solution on four artificial flowers placed in their home range. After thirty consecutive foraging bouts, all the hornets had developed a repeatable flower visitation sequence. Using two different arrays of flowers, we also show that hornets consistently increased their foraging efficiency with experience. However, they did not always use the shortest path to visit all the flowers, often favoring movements between nearest-neighbour options rather than minimizing overall travel distance. Our study thus adds nectar-foraging wasps to the list of animals that exhibit trapline foraging, thereby significantly broadening the scope for comparative research in multi-destination route learning and memory.

**Supplementary Information:**

The online version contains supplementary material available at 10.1007/s10071-025-01952-3.

## Introduction

Animals feeding on plant nectar and pollen must visit dozens to thousands of flowers within a single foraging trip in order to reach their nutritional needs. In species with a stable home range, such as bees, butterflies, hummingbirds, and many bats, individuals tend to develop repeatable foraging circuits to exploit known food locations that renew over time (Janzen 1971; Boggs et al. 1981; Ohashi et al. 2009; Lihoreau et al. 2013; Tello-Ramos et al. 2015). This complex routing behaviour, known as “trapline foraging” (Thomson et al. 1997), is thought to provide substantial benefits in terms of reduced competition and increased rate of energy gain (Possingham 1989).

The development of traplines has been described in detail through the observation of individual foragers exploiting arrays of feeders (i.e. artificial flowers) over several consecutive hours. For instance, bumblebees and honey bees foraging alone in an array of four to ten artificial flowers with high rates of nectar renewal tend to find the shortest route to visit all flowers once and return to their nest (Buatois et al. 2024). While foragers initially tend to use simple movement rules, such as visiting nearest unvisited neighbour flowers, spatial memories and information associated with nectar quantity and refilling rate may further enable route optimization (Ohashi et al. 2009; Lihoreau et al. 2013). Hence, through trial and error, foragers then gradually reduce their revisits to empty flowers, use straight flight paths between visiting flowers, and arrange their flower visitation sequences in such a way that they minimize overall travel distances (Lihoreau et al. 2012a; Reynolds et al. 2013). Once established, a trapline can last for days, or even weeks, provided that food resources remain stable and replenish (Thomson et al. 1996).

In bees, route optimization depends on the spatial distribution of resources. For instance, foragers are more efficient at using traplines minimizing travel distances at large spatial scales when feeding sites are spaced by dozens or hundreds of meters (i.e. between flower patches) than at small spatial scales when feeding sites are spaced by only few centimeters or meters (i.e. within a flower patch) (Lihoreau et al. 2010, 2012b). Presumably, at such small spatial scales, the energy cost of using a long suboptimal route is negligible compared to the cognitive cost involved in route learning and optimization (Buatois and Lihoreau 2016). At both spatial scales, however, travel optimization is often imperfect, as bees tend to favour straight movements over sharp turns when moving between flowers (Ohashi et al. 2007; Woodgate et al. 2017), as well as jumps to nearest flowers over longer strategic bypasses (Saleh and Chittka 2007).

Since many nectarivores mediate pollen dispersal, plant mating patterns, and ultimately plant fitness, understanding the breadth of trapline foraging across the animal kingdom as well as commonalities and differences between species holds considerable promise for better comprehending plant–pollinator interactions from a mechanistic point of view and informing precision pollination (Mailly et al. 2024, 2025). For instance, accurate prediction of flower visitation sequences by pollinators can be used to make new hypotheses about pollen dispersal, pollination success, and plant fitness in a context of a looming pollination crisis (Ohashi and Thomson 2009).

Here we explored whether the development of such multi-destination routes could also be observed in central place foraging nectarivore wasps. Just like honey bees and bumblebees, social wasps must collect large quantities of floral nectar to provision their colonies (Bouchebti et al. 2022). Previous studies using mark-recapture approaches have shown that Asian yellow-legged hornets (*Vespa velutina*) often return to the same foraging places for hunting and collecting nectar (Monceau et al. 2014; Ueno 2015), suggesting they can aquire multiple place memories and have the ability to develop traplines. We tested this ability in a population of wild Japanese yellow hornets *Vespa simillima* foraging in two different arrays of four artificial flowers in the field.

## Methods

### Study site and insects

**Fig. 1:**
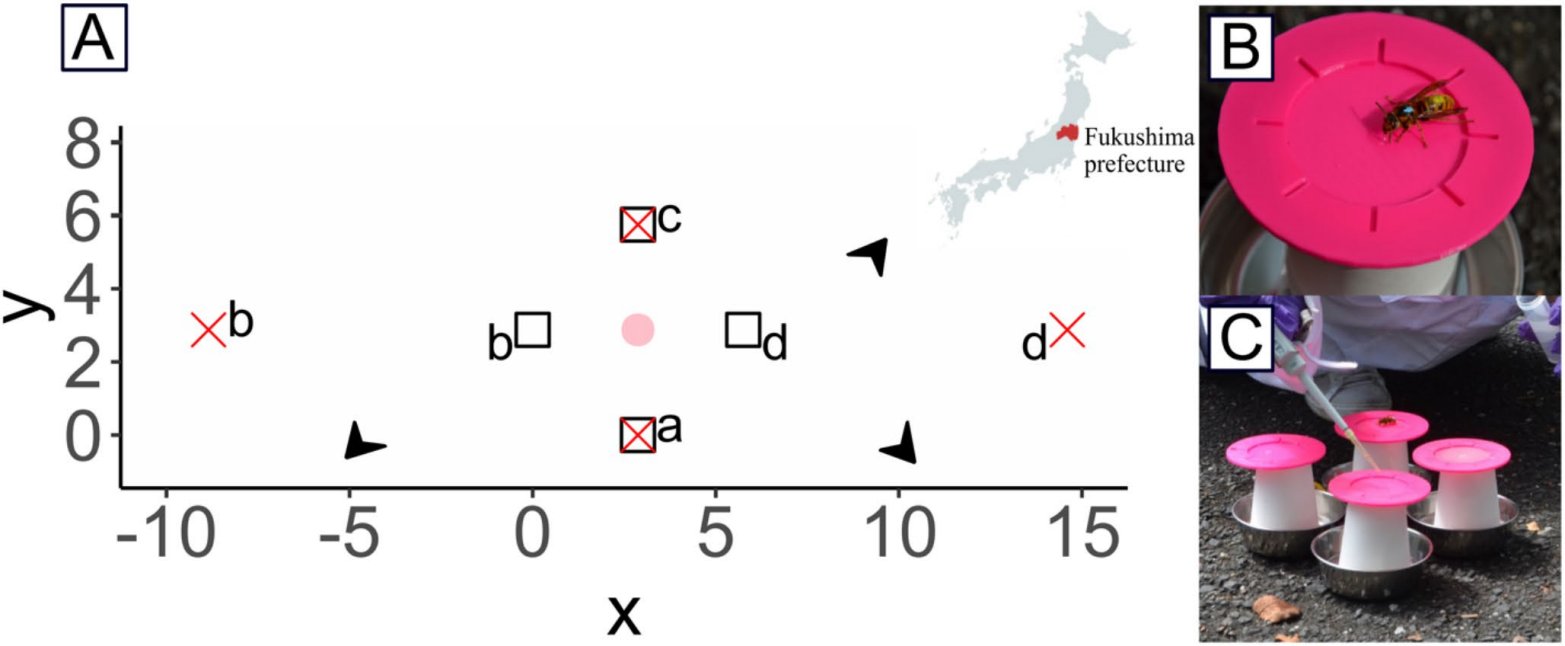
Arrays of artificial flowers. (**A**) We settled artificial flowers (a-d) on a private road in the middle of our experimental site in the Fukushima prefecture, Japan (37°37’35.612’’N, 140°54’17.567’’E, map produced with MapChart). The pink disk highlights the pre-training location. The small array of flowers is depicted with white squares, with flower coordinates in meters (x, y): a(2.875, 0), b(0, 2.875), c(2.875, 5.750), d(5.750, 2.875). The large array of flowers is depicted with red crosses, with flower coordinates in meters: a(2.875, 0), b(-8.850,2.875), c(2.875, 5.750), d(14.600, 2.875). Black arrows indicate the directions of the different hornet nests from the training location (precise nest locations are unknown). (**B**) Picture of a paint-marked hornet collecting sucrose solution on an artificial flower. (**C**) Picture of the patch of four flowers used for pre-training. (Credit for pictures in B and C: Jean-Marc Bonzom/ARSN)

We conducted the experiments at a study site located in Minamisoma city (Fukushima prefecture, Japan: 37°37’35.612’’N, 140°54’17.567’’E) in September 2023. We worked on a closed road bordered by cherry tree plantations in which six honey bee hives (*Apis mellifera*) had been placed in May 2023. At this period of the year, a population of wild yellow hornets (*V. simillima*) was predating on the honey bees. We attracted some of these hornets to artificial food sources by placing a gravity feeder containing *ad libitum* 40% (v/v) sucrose solution in the middle of the road (see pre-training location in Fig. 1). We then marked the hornets while they were collecting sucrose on the feeder by applying a drop of enamel paint on their thorax, abdomen, or both, for individual recognition. After feeding, the hornets flew away in straight lines towards their colony nests. Detailed observations of these return flights indicated that there were three different hornet nests around the study site (see arrows in Fig. 1).

### Pre-training

Once we had more than five hornets frequently visiting the feeder, we began the pre-training. For this, we caught all the hornet foragers in individual cages (plastic cups) to avoid aggressive interactions between them that could interfere with their natural foraging behaviour during the experiment (personal observations). We then randomly selected one of these hornets (i.e. the focal individual) and released it for testing. This hornet was presented four identical feeders (hereafter called “artificial flowers”) arranged in a patch at the pre-training location (flowers were interspaced by 5 cm; Fig. 1A) on the road.

Each flower was made of a large 3D-printed pink plastic landing platform (diameter 10 cm) placed on top of a transparent cylindrical paper cup (diameter 5 cm, height 8 cm; Fig. 1B). We chose pink as this colour is visible to many hymenopterans (Briscoe and Chittka 2011) and we could not find it on natural flowers in the study area, thereby ensuring that the trained hornets would only visit our artificial flowers during the experiment. The spectral reflectance of the pink polyactic acid filament (RS Components) used to produce the flowers is available in Fig. S1. The flower was placed in a metallic cup (diameter 12 cm, height 3 cm) filled with water so that ants could not climb the platform and feed on the sucrose reward. The complete device (i.e. flower and its support) sat directly on the ground.

We measured the nectar crop capacity of the focal hornet (i.e. maximum amount of liquid the hornet could collect to fill its crop to capacity) by presenting 10µL drops of 40% sucrose solution with a micropipette (Genex beta P20) in the middle of the landing platform of each flower, and refilling them with new drops of the same volume each time the hornet collected them (Lihoreau et al. 2010) (Fig. 1C). In this way, we monitored the total volume of nectar collected by the hornet during a single foraging bout (i.e. foraging sequence starting when the hornet came to visit the array of flowers and ending when the hornet flew back to its nest) over five consecutive foraging bouts, which enabled us to estimate the crop capacity of each hornet by averaging the volume collected during the five foraging bouts (min = 152 µl, max = 360 µl, *N* = 8 hornets).

### Training

Once the crop capacity of the focal hornet was estimated and the hornet had returned to its nest, we moved the four artificial flowers into training positions (see flower coordinates in Fig. 1A). We used two different arrays of flowers to discriminate between potential route optimization strategies.

In the “small array”, the four flowers were arranged in a square shape of 4 m side. A hornet moving between nearest-neighbour flowers would use the shortest possible route to visit all flowers at once. In the “large array”, the four flowers were arranged in a diamond shape of 12 m side, creating a conflict between different spatial strategies. Here a hornet moving between nearest-neighbour flowers would use a long, suboptimal, route (i.e. traveling the optimal route would involve circulating around the array). The spatial scales used in our study were comparable to those of previous reports on bees (Ohashi et al. 2007; Lihoreau et al. 2010, 2012b; Buatois and Lihoreau 2016; Buatois et al. 2024), thereby facilitating comparisons across studies and species. Although the hornets had to travel three times longer to visit all flowers once in the diamond shape array (shortest possible route: 36 m) than in the square shape array (shortest possible route: 12 m), the two spatial scales remained small enough to make us expect comparable optimization behaviour based on the bee studies (Buatois and Lihoreau 2016).

For both arrays of flowers, at the beginning of a foraging bout, each flower contained 1/4th of the nectar crop capacity of the focal hornet, so that the insect had to visit the four flowers to fill its crop to capacity before returning to its nest. We tested each focal hornet for 30 consecutive foraging bouts in a given array (average 113.15 ± (SD) 26.50 min of observations per hornet, *N* = 8 hornets).

We collected the data through visual observation using a custom-made computer program enabling to record events (time of occurrence of a given behaviour) by pressing a key on a computer keyboard (Lihoreau et al. 2010). At each foraging bout of each hornet, we recorded the time at which the hornet landed and took off from each flower, as well as the time at which the hornet returned to its nest. Each hornet was tested on a different day. After testing a focal hornet, we released all the captive hornets, previously trained and individually marked, for future tests.

In total, we tested 5 hornets in the small array and 3 hornets in the large array. These relatively small sample sizes are explained by the difficulties of conducting field work on wild species during the typhoon season in Japan (September). However, we collected enough data for a qualitative description of trapline foraging, in line with previous studies addressing similar questions in bees (Lihoreau et al. 2010, 2011, 2012a, b; Buatois and Lihoreau 2016; Buatois et al. 2024; Ohashi et al. 2007; Saleh and Chittka 2007; Woodgate et al. 2017).

### Data analysis

We performed all the statistical analyses with R and produced figures and tables with R and Python. Before any analysis, we excluded from the raw data all revisits to flowers after the 4 flowers of the array have been visited within the same foraging bout (i.e. when all flowers were emptied; Lihoreau et al. 2010). These revisits occurred in less than 5% of all the foraging bouts and were not informative about trapline formation.

**Fig. 2:**
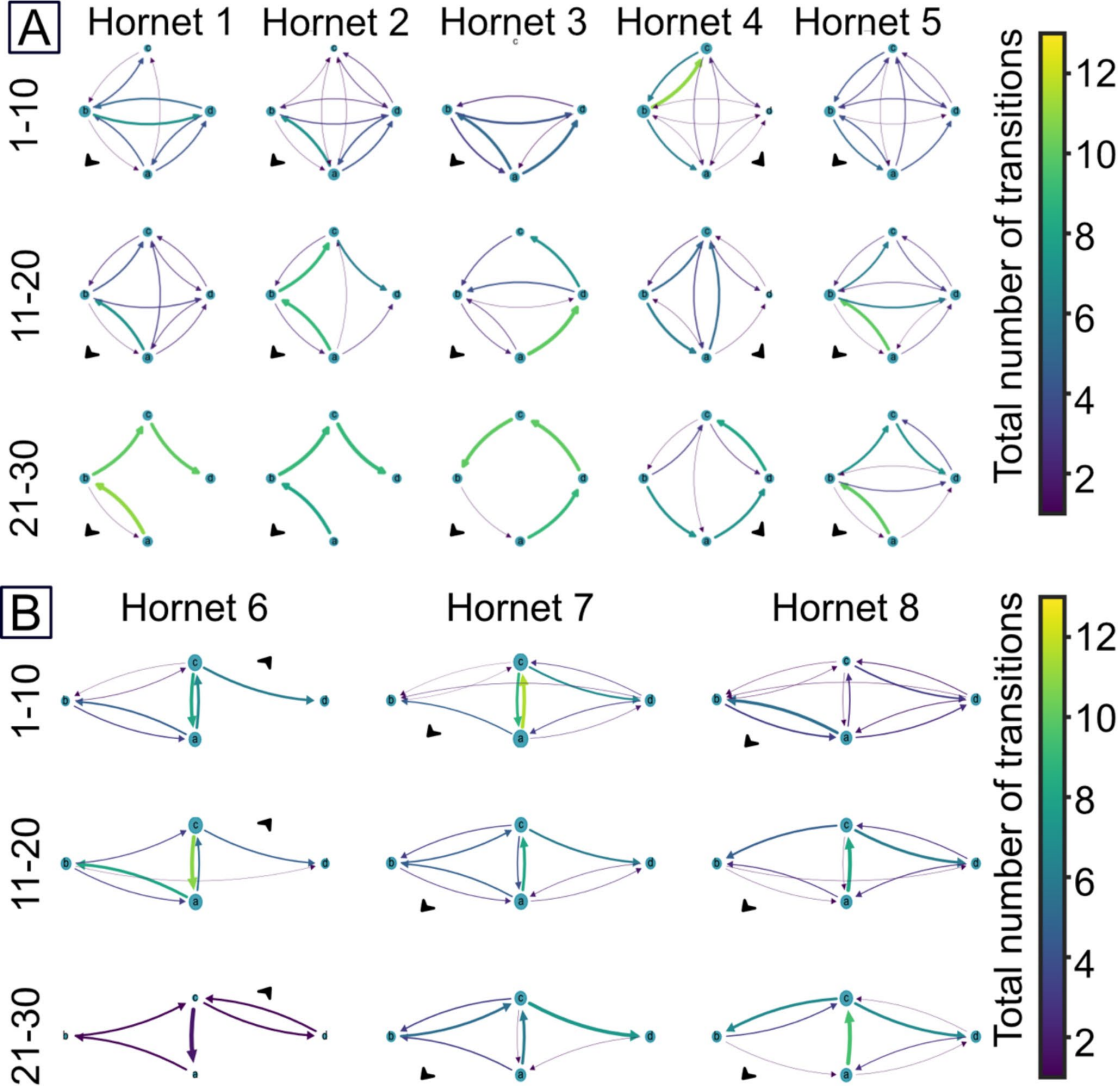
Aggregated spatial movement networks of individual hornets (**A**) in the small array of flowers and (**B**) in the large array of flowers. Columns show data for individual hornets. Rows represent bins of 10 foraging bouts (hornet 6 only completed 22 foraging bouts, see details in Fig. S2). Blue nodes represent flowers (see details about flower names and coordinates in Fig. 1A). The diameter of each node is proportional to the number of visits on those flowers for each bin of 10 foraging bouts. The thickness of each arrow is proportional to the total number of executed transitions for each bin of 10 foraging bouts. The direction of the arrows indicates the direction of the movement between flowers. The absolute number of transitions is indicated by the colormap. Black arrows indicate the directions of the nest of each hornet

To visualize hornet movements, we plotted spatial movement networks (Fig. 2) illustrating all the transitions between artificial flowers by hornets using NetworkX Python (Hagberg et al. 2008). For a given hornet, these networks represent the frequency and direction of all movements between two flowers (Pasquaretta et al. 2019).

We then assessed the repeatability and the optimality of flower visitation sequences for each foraging bout of each hornet using four metrics: (1) the determinism index (DET), (2) the revisitation rate, (3) the crop filling rate, and (4) the relative difference between travelled distance and optimal route (see details below).

We analysed route repeatability by calculating the determinism (DET), following methods described by Ayers et al. (2015) and Buatois and Lihoreau (2016). The DET indicates how much an animal repeats sequences of visits to multiple locations through time: an index of 1 indicates a perfectly repeated sequence of visits, while an index of 0 indicates the complete absence of repetition. The DET takes into account sequence length and revisits as well. Here, we ignored sequences that included less than three visits. Sequences were considered similar when they followed exactly the same order, i.e. the route linking flowers a-b-c-d was different from the route d-c-b-a. We grouped the foraging bouts of each hornet into bins of five bouts in order to measure the DET, assess its dynamics through time, and the impact of the flower array size. As a control, we computed the average DET obtained from 100 simulated random flower visitation sequences (i.e. random choice among four flowers, until all flowers have been visited at least once).

We assessed foraging efficiency by computing revisits to empty flowers during the same foraging bouts. We considered two types of revisits (Lihoreau et al. 2010). The rate of immediate revisits corresponded to the number of times a hornet returned to a flower without visiting any other flowers in between. The rate of non-immediate revisits corresponded to revisits to a flower after having visited other flowers.

We also calculated the nectar crop filling rate, assuming the hornet’s crop capacity to be filled by 25% each time it visited a flower containing a sucrose reward (the precise volume of nectar collected by a hornet during a flower is unknown). We thus calculated the nectar crop filling rate as the number of visits to flowers containing a reward divided by the total time spent per foraging bout.

We estimated route optimality by estimating travel distances during each foraging bout. We assumed that hornets flew straight lines between their nest and flowers, and between flowers, and summed all these distances for each foraging bout (Lihoreau et al. 2010). We approximated the nest positions by placing them in a theoretical circle whose center is in the middle of the flower arrays and has a 20 m radius. We then computed the relative difference between the observed travel distance (i.e. sum of all straight lines between flowers visits of a foraging bout) and the optimal distance (i.e. shortest route to visit all flowers once) divided by the optimal distance multiplied by 100. This metric indicated the deviation from optimal. A deviation of 0% means that the hornet used the optimal route.

We analysed changes of these four metrics through time (foraging bout number) using Generalized Linear models (GLM). Due to the small sample size (5 and 3 hornets tested in each flower array) the random effects were not added as we had less than 5 grouping levels and not enough observations (Gelman and Hill 2006). We removed outliers for the crop filling following the interquartile rule (we removed observations that fall below Q1 − 1.5 IQR or above Q3 + 1.5 IQR).

Flower visitation sequences of each hornet (excluding immediate revisits) are shown in Fig. S2. Complete dataset is available in the Dataset S1.

## Results

### Small flower array

We first tested whether hornets could develop traplines in a small array of flowers (4 m side square) in which movements between nearest neighbour flowers would lead to the shortest possible route (Fig. 1). Visual inspection of the spatial movement networks, computed on bins of ten foraging bouts, indicates that hornets used increasingly repeatable and efficient flower visitation patterns through time. This is illustrated by the arrows that tended to be less numerous, larger and brighter, and the nodes that tended to be of similar size in the last bin of foraging bouts than in the first bin (Fig. 2A).

**Fig. 3:**
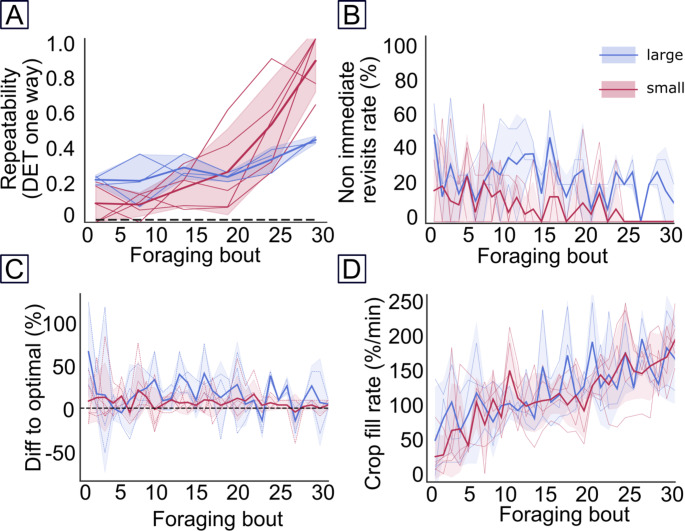
Foraging performances of hornets in the small (red, *N* = 5 hornets) and the large (blue, *N* = 3 hornets) array of flowers over 30 consecutive foraging bouts. (**A**) Route repeatability as measured by the determinism index (DET). The black dotted line represents the expected DET expected from random movement (DET = 0.014 ± 0.07, *N* = 100 simulated sequences). (**B**) Number of non-immediate revisits to empty flowers (in percentage). (**C**) Relative difference between traveled distance and optimal route minimizing travel distances (in percentage). (**D**) nectar crop filling rate (in percentage per minute). Solid lines represent mean. Coloured areas are standard deviations. Coloured dashed lines represent the individual data. In C) the black dashed line represents the optimal route minimizing overall travel distance. Data under this line means individuals did not visit the 4 flowers

All 5 hornets tested increased their route repeatability as they gained experience with the array of flowers (DET: GLM with gaussian distribution, df = 43, t = 8.682, p-value < 0.001; Fig. 3A). This was accompanied by a reduction in their number of immediate and non-immediate revisits to empty flowers (GLM gamma distribution with an inverse link function, immediate revisits: df = 228, t = 5.755, *p* < 0.001; non-immediate revisits: df = 221, t = 4.35, p-value < 0.001, Fig. 3B), as well as a reduction of the deviation between estimated travel distances and the shortest possible travel distance (difference to optimal: GLM gamma distribution with an inverse link function, df = 132, t = 3.00, p-value < 0.001; 2.49 ± 10.24% in the last 5 foraging bouts; Fig. 3C). Consequently, the hornets significantly increased their crop filling rate through time (GLM gaussian distribution, df = 221, t = 8.036, *p* < 0.001, Fig. 3D).

On average, the hornets used the shortest possible flower visitation sequence in 51.33% ± 19.63 (*N* = 5) of all their foraging bouts, moving between nearest unvisited flowers in 72.02 ± 6.85% of all their transitions, from 50.14 ± 6.03% in their first ten foraging bouts, to 91.40 ± 8.29% in their last ten foraging bouts. Four out of the 5 hornets used the shortest possible route in at least 7 of their 10 last foraging bouts, indicating that they stabilized this optimal route towards the end of training (see flower visitation sequences of hornets 1,2,3,5 in Fig. S2).

Interestingly, the start position and direction of rotation of the flower visitation sequences varied greatly across individual hornets (see Fig. S2). Each hornet began their flower visitation sequence by landing on the same flower in 87.5% ± 4.61 (*N* = 5) of all their foraging bouts. In 4 out of the 5 hornets, this flower was different from the very first flower they discovered during the first foraging bout, indicating that hornets did not simply visit flowers in their discovery order. This flower was different across hornets and was likely chosen because it was close to their nest location. Hornets also showed some inter-individual differences in their direction of rotation in the array, with 3 hornets visiting the flowers by turning clockwise while 2 hornets turned anti-clockwise. This high level of variation in movement patterns further indicates that that route establishment was the consequence of individual learning and memory.

### Large flower array

We next tested whether hornets could still develop traplines in a larger array (12 m side diamond) in which movements between nearest neighbour flowers would lead to a long suboptimal route (Fig. 1). Here again the visual inspection of the spatial movement networks computed on bins of ten foraging bouts suggests that hornets developed more stable and efficient routes through time as illustrated by less numerous, brighter and larger transitions between flowers and the more similar node sizes in the last bin than in the first bin of foraging bouts (Fig. 2B).

The three hornets tested did not significantly increase their route repeatability with experience (DET, GLM with gaussian distribution, df = 43, t = 1.525, p-value = 0.13; Fig. 3A). They also did not significantly reduce their number of non-immediate revisits through time (GLM with gamma distribution, non-immediate revisits: df = 221, t = 0.864, p-value = 0.39; Fig. 3B). However, they significantly increased their foraging efficiency as indicated by the reduction of their number of immediate revisits to flowers (GLM with gamma distribution, immediate revisits: df = 228, t = 4.063, *p* < 0.001), the decrease of the deviation between estimated travel distances and the shortest possible travel distance (difference to optimal: GLM gamma distribution with an inverse link function, df = 132, t = 2.59, *p* = 0.01; 16.72 ± 23.13% in the last 5 foraging bout; Fig. 3C), and the increase of nectar crop filling rate (crop fill rate: GLM, df = 221, t = 5.47, p-value < 0.001, Fig. 3D).

In this larger array of flowers, the hornets used the shortest possible sequence to visit all flowers in only 30.38% ± 11.25 of their foraging bouts (see detailed sequence visitation in Fig. S2). Instead, they favoured movements between nearest unvisited neighbour flowers in 59.35 ± 3.92% of all their transitions, from 51.46 ± 4.11% in their first ten foraging bouts, to 76.97 ± 9.50% in their last ten foraging bouts. Thus, in contrast to in the small array of flowers, the hornets did not stabilize the shortest possible route towards the end of training (see visitation sequences of hornets 6,7,8 in Fig. S2).

However, here again, the beginning of the flower visitation sequence and the direction in which the hornets turned in the array varied greatly among individuals (see Fig. S2). Each individual began their flower visitation sequence by landing on the same flower in 81.1% ± 7.3 (*N* = 3). In 2 out of the 3 hornets, this flower was different from the very first flower they discovered during the first foraging bout and was close to the nest location, further indicating that the hornets rearranged their flower visitation sequences as they gained experience with the array.

When comparing the behaviour of hornets in the two flowers arrays, our results suggest poorer route development performances in the large array compared to the smaller one (DET: df = 43, t = -3.857, p-value < 0.001; non-immediate revisits: df = 221, t = 3.69, *p* < 0.001). However, the overall foraging efficiency, estimated by the crop filling rate and the relative deviation to the optimal route, was similar in both conditions (crop filling rate: df = 221, t = -0.17, p-value = 0.867, difference to optimal: df = 132, t = 1.09, *p* = 0.278).

## Discussion

Bees, butterflies, birds, bats and primates that feed on renewable plant resources within a stable home range use traplines to efficiently exploit plant resources in their environment (Janzen 1971; Boggs et al. 1981; Racey and Swift 1985; Ohashi and Thomson 2009; Noser and Byrne 2010; Lihoreau et al. 2013; Tello-Ramos et al. 2015). Here, for the first time, we report that a nectar-collecting wasp, the Japanese yellow hornet, also develops traplines to forage on artificial flowers. This observation supports the idea that trapline foraging is a widespread adaptation to flower foraging (Rombault et al. 2022) and offers new opportunities to explore how animals with so different biologies, ecologies and central nervous systems solve a common multilocation problem.

Trapline foraging by hornets was most evident in the small array of flowers, when nectar sources were arranged in a square configuration of 4 m side. In this environment, all hornets gradually developed a route to visit all flowers once and return to their nest, by visiting flowers in clockwise or anti-clockwise order. Route development was accompanied by a reduction of revisits to empty flowers and travel distances. Hornets thus increase their overall foraging efficiency through time, sometimes leading to the stabilization of the shortest possible route. This observation is very much comparable to previous reports in bumblebees and honey bees collecting nectar on artificial flowers at similar spatial scales (Lihoreau et al. 2010, 2011; Buatois and Lihoreau 2016).

The hornets also reached high foraging efficiency in the larger flower array, when nectar sources were arranged in a diamond configuration of 12 m side. The insects tended to make fewer revisits to empty flowers, increased their crop filling rate and reduced the difference between actual travelled distance and the shortest possible route as they gained experience in the array. In this condition, none of them stabilized a route. However, non-random levels of route repeatability were still reached by favouring movements between nearest-neighbour flowers, forcing them to detour from the shortest possible flower visitation sequence. This observation is also consistent with previous studies in bees reporting that bumblebee foragers favour movement between nearest neighbours over sharp turns, even if this leads to suboptimal routes in flower arrays where nearest-neighbour movements and travel optimization are put in conflict (Ohashi et al. 2007; Woodgate et al. 2017). Interestingly, and as expected from these bee studies, hornets from different nests initiated their route by visits to flowers that were closest to their nest, highlighting the importance of nest location in the final geometry of traplines.

Note that the absence of travel optimization by hornets in our large array of flowers does not demonstrate an inability to do so. Several reasons could explain this result. Firstly, it is possible that travel distances in the experiment were too small to incur significant movement costs, even in our large flower array, and to trigger a route optimization behaviour. Bees, for instance, are more prone to optimize their route geometry at large spatial scales, when feeding sites are spaced by several dozen meters and using long suboptimal routes is energetically costly than in conditions when feeding sites are aggregated (Lihoreau et al. 2012a; Buatois and Lihoreau 2016). Japanese yellow hornets likely have large foraging areas, compared to that of bumblebees and honey bees, as suggested by observations on similar sized yellow-legged hornets (*Vespa velutina)* that were observed to forage up to at least five kilometers around their nest (Poidatz et al. 2018) and possess a maximum flight capacity of dozen kilometers (Sauvard et al. 2018). The small difference of only a few meters between the longest and the shortest possible routes in our large experimental array of flowers could therefore be negligible regarding to the flight capacities of hornets that may frequently travel larger distances to visit many more feeding sites per foraging bout.

It is also possible that the duration of our observations was not long enough (30 trials, range: 76 min 47s − 158 min 5s) in order to enable hornets to find and stabilize the shortest possible route in our setups. In bumblebees, the number of trials necessary for foragers to develop and optimize a route greatly varies with spatial scale and arrangement of artificial flowers (e.g. 20 trials at large spatial scales in Lihoreau et al. 2010, 80 trials at small spatial scales in Lihoreau et al. 2011). Finally, our observations are based on a relatively small number of hornets due to field work difficulties, and we cannot exclude important interindividual variability in these spatial behaviours, as already reported in bumblebees (Klein et al. 2017). Future experiments should therefore explore the spatial foraging patterns of hornets on larger sample sizes, in a wider range of spatial scales, numbers of feeding sites, and training durations.

Our observations of trapline foraging in Japanese yellow hornets indicate that these insects use spatial and visual memories when foraging for nectar, and highlight a strong resource fidelity. Whether central place foraging hornets, like many species of bees, develop traplines between natural nectar resources and between the same flower species (flower constancy) is an open question whose answer will enable better assessment of their contribution to pollination in a context where they also represent a threat for many pollinators (Brock et al. 2021; Rojas-Nossa et al. 2023).

## Electronic supplementary material

Below is the link to the electronic supplementary material.

Supplementary Material 1

Supplementary Material 2

## Data Availability

Data is provided in supplementary information files.
