# Supplementary material for: Trapline foraging by nectar-collecting hornets
Source: Anim Cogn. 2025 Apr 19;28(1):30. doi: 10.1007/s10071-025-01952-3 (PMC12009247; doi:10.1007/s10071-025-01952-3)
Supplement: Supplementary file 2 — Supplementary Material 2 [file 10071_2025_1952_MOESM2_ESM.docx]

**Supplementary materials**

**
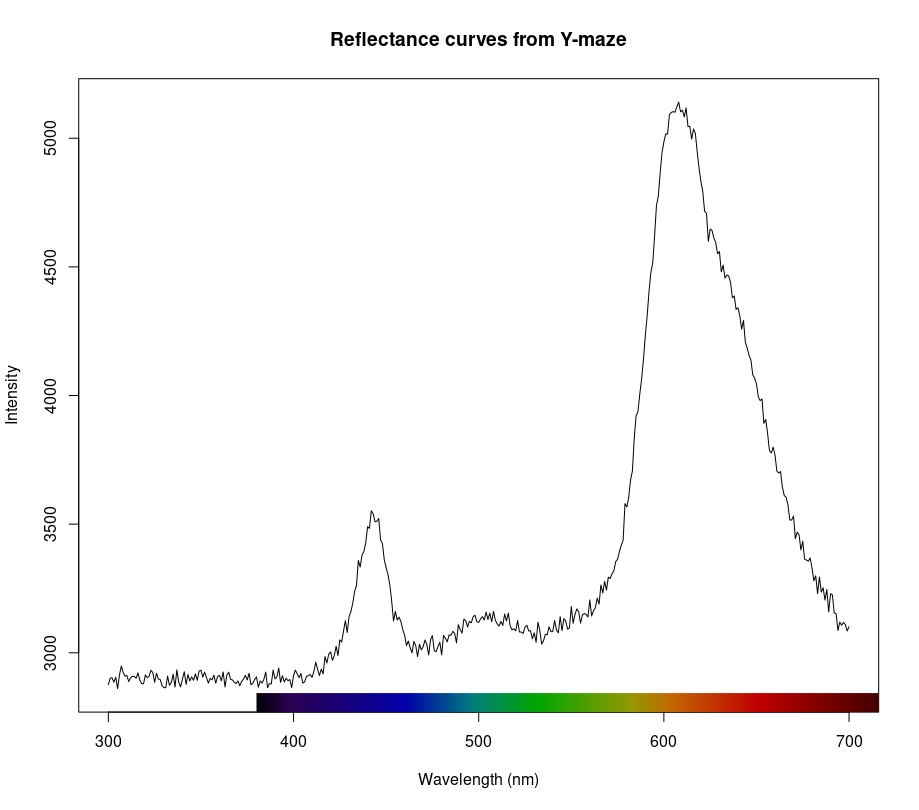
Fig S1.** Spectral reflectance of the pink plastic used to make the landing platforms of the artificial flowers (measured with a FLAME-T-UV-VIS spectrophotometer) The pink has two peaks at 442 nm and 608 nm.


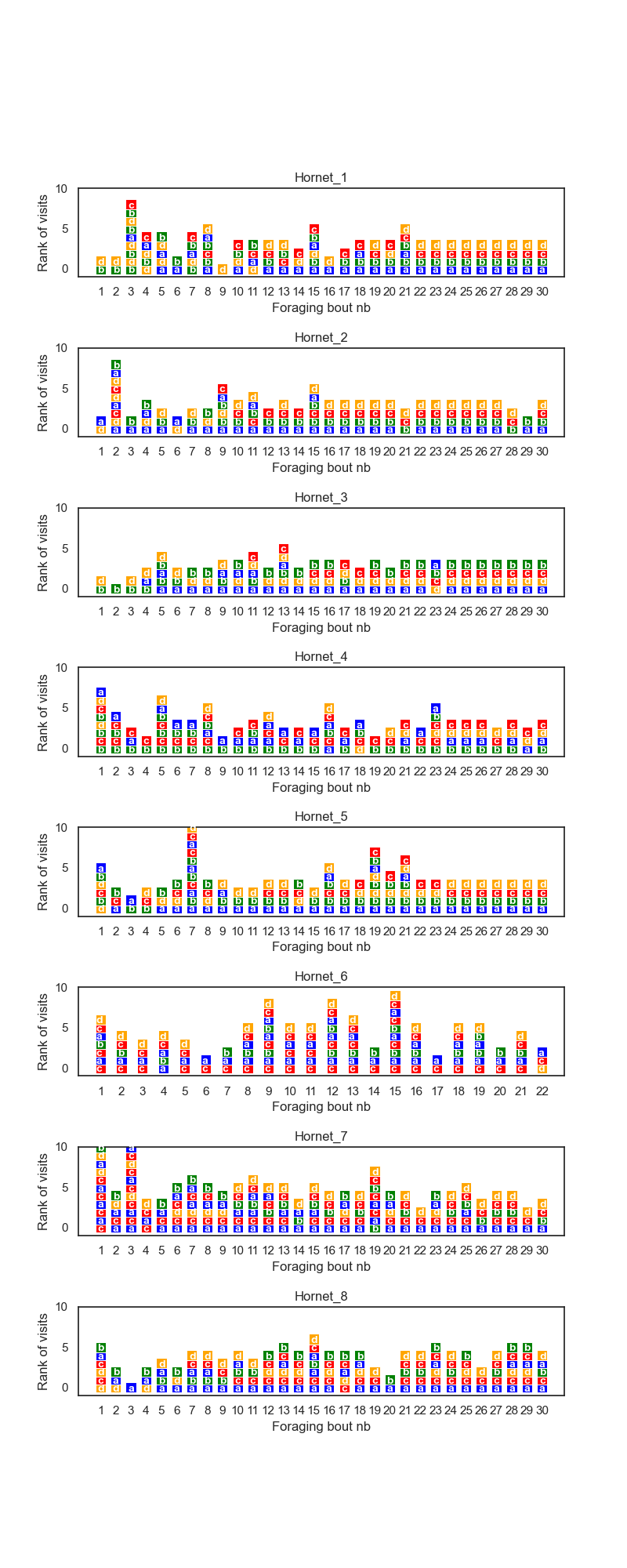


**Fig S2.** Flower visitation sequences by hornets excluding immediate revisits. Coloured letters represent single visits to different flowers (see details about the spatial arrangements of flowers in Fig. 1A).

**Dataset S1.** Csv file compiling all flower visitation sequences for all hornets.
